# Viscosity-adjustable grease matrices for serial nanocrystallography

**DOI:** 10.1038/s41598-020-57675-7

**Published:** 2020-01-28

**Authors:** Michihiro Sugahara, Koji Motomura, Mamoru Suzuki, Tetsuya Masuda, Yasumasa Joti, Keiji Numata, Kensuke Tono, Makina Yabashi, Tetsuya Ishikawa

**Affiliations:** 1 RIKEN SPring-8 Center, 1-1-1 Kouto, Sayo-cho, Sayo-gun, Hyogo 679-5148 Japan; 2 Institute for Protein Research, Osaka University, 3-2 Yamadaoka, Suita, Osaka 565-0871 Japan; 3 Division of Food Science and Biotechnology, Graduate School of Agriculture, Kyoto University, Gokasho, Uji, Kyoto 611-0011 Japan; 4 Japan Synchrotron Radiation Research Institute, 1-1-1 Kouto, Sayo-cho, Sayo-gun, Hyogo 679-5198 Japan; 5 Biomacromolecules Research Team, RIKEN Center for Sustainable Resource Science, Hirosawa, Wako-shi, Saitama 351-0198 Japan

**Keywords:** Proteins, Nanocrystallography

## Abstract

Serial femtosecond crystallography (SFX) has enabled determination of room temperature structures of proteins with minimum radiation damage. A highly viscous grease matrix acting as a crystal carrier for serial sample loading at a low flow rate of ~0.5 μl min^−1^ was introduced into the beam path of X-ray free-electron laser. This matrix makes it possible to determine the protein structure with a sample consumption of less than 1 mg of the protein. The viscosity of the matrix is an important factor in maintaining a continuous and stable sample column from a nozzle of a high viscosity micro-extrusion injector for serial sample loading. Using conventional commercial grease (an oil-based, viscous agent) with insufficient control of viscosity in a matrix often gives an unexpectedly low viscosity, providing an unstable sample stream, with effects such as curling of the stream. Adjustment of the grease viscosity is extremely difficult since the commercial grease contains unknown compounds, which may act as unexpected inhibitors of proteins. This study introduces two novel grease matrix carriers comprising known compounds with a viscosity higher than that of conventional greases, to determine the proteinase K structure from nano-/microcrystals.

## Introduction

Serial femtosecond crystallography (SFX) using X-ray free-electron lasers (XFELs) with ultrashort X-ray pulses has enabled determination of structures of protein crystals without influences of an undesired radiation damage via the “diffraction-before-destruction” approach^1–6^. A number of crystal structures of interesting proteins at room temperature have been determined through this technique^7–18^. For serial sample loading, an injection by a liquid jet containing small protein crystals, with a continuous flow at a relatively high speed of ~10 m sec^−1^ has been exploited^19^, at least consuming 10 mg–100 mg of the protein for crystals. To reduce the protein sample consumption in SFX, micro-extrusion methods of specimens using viscous carrier media, such as a lipidic cubic phase (LCP)^10,20^, grease^11,21,22^, Vaseline (petroleum jelly)^23^ and agarose^24^ have been developed. Recently, a micro-extrusion method using viscous media, such as hyaluronic acid^21^, hydroxyethyl cellulose^22^, sodium carboxymethyl cellulose^25^, the thermo-reversible block polymer Pluronic F-127^25^, a high-molecular-weight poly(ethylene oxide) (PEO)^26^ and a polyacrylamide^27^, have enabled maintaining a stable stream at a low flow rate of 0.02 μl min^−1^–0.6 μl min^−1^, which helps in reducing sample consumption to less than ~1 mg. Together with XFEL experiments, serial crystallography has also been developed even at synchrotron facilities^23,25,26,28–32^, demonstrating an importance of the sample loading technique with viscous media in serial crystallography. Although the technique using viscous carrier media is technically simple, some media produce stronger X-ray scatterings to induce an increase in the background noise level^21,22^. A crystal carrier with low background scattering is essential for data collection from small crystals (~1 μm or less), at atomic resolution and *de novo* phasing with weak anomalous signals, to improve the signal-to-noise ratio^24^. Furthermore, it would be important to prepare a wide repertoire of carrier media which should be provided for a wide variety of proteins, to prevent crystal damage induced by the matrix.

In 2015, Sugahara *et al*.^11^ successfully introduced a mineral oil-based grease as a versatile crystal carrier in SFX at SACLA. This medium, however, created very strong background scattering. To reduce background scattering generated from the carrier media, we have recently introduced hyaluronic acid^21^ and hydroxyethyl cellulose (cellulose matrix)^22^ in SFX. By introducing this cellulose matrix in SFX, the structure of proteinase K was successfully determined at an atomic resolution of 1.2 Å^33^. The application of oil-free, viscosity-adjustable hydrogel matrices for use in SFX has now been expanded to grease-sensitive proteins. In addition, commercial grease matrices with low background scattering—Super Lube synthetic grease and Super Lube nuclear grade grease (nuclear grease)—were also introduced into SFX studies at SACLA^21,22^. To date, grease matrices have been demonstrated to be highly adaptable in SFX using a wide variety of soluble and membrane proteins: lysozyme, glucose isomerase, thaumatin, fatty acid–binding protein type 3 or proteinase K^11,21,22,34^, copper nitrite reductase^35^, photosystem II^15^, luciferin-regenerating enzyme^36,37^, the photo-switchable fluorescent protein IrisFP^13^, bacteriorhodopsin^38^, bacterial phytochrome^39^, thermolysin^40^, the stem domain of human POMGnT1^37^, galectin^37^, the photochromic fluorescent protein nonlinear structured illumination variant mEos3.1H62L (Skylan-NS)^41^ and the non-photosynthetic fruiting myxobacterium, *Stigmatella aurantiaca* SaBphP1-PCM (the complete photosensory core module)^42^. Thus, readily available and applicable grease matrix should be vital and required in SFX, however, the viscosity of commercial grease would not be sufficiently controlled with considerable variation among different manufacturing lots. A low viscosity matrix tends to produce both curling of the stream and a grease column with an increased diameter relative to the injector nozzle i.d. These phenomena are particularly notable when the grease matrix is blown off by XFEL pulses, attaching the matrix oil to the injector nozzle top. Since columns with a small diameter (for instance, 50 μm) are substantially affected by low viscosity, it required to increase the matrix viscosity, by removing as much of the supernatant (harvest) solution in the crystal solution as possible before mixing the matrix with crystals, or by decreasing the crystal density in the matrix. The adjustable matrix viscosity, however, had an upper limit. Additionally, the components of compounds in commercial grease are mostly unspecified; some may act as unexpected inhibitors of protein activity. For serial sample loading, a viscosity-adjustable grease comprising known materials is required to maintain a stable sample stream.

In this article, we introduce two viscosity-adjustable grease matrices—dextrin palmitate/liquid paraffin grease and dextrin palmitate/dialkyl tetraphenyl ether (DATPE) grease into SFX. The structures of proteinase K from *Engyodontium album* were determined by using nano- and microcrystals (0.8 μm × 0.8 μm and 2 μm × 2 μm, respectively). We also investigated the effects on the thawing of frozen proteinase K microcrystals which had dispersed in the grease matrix. Since the improvement as well as efficiency of sample preparation is crucical in the SFX study, this approach would be helped in overcoming the time consuming for sample preparation and become more versatile method.

## Results

### Micro-extrusion of microcrystals

**Figure 1:**
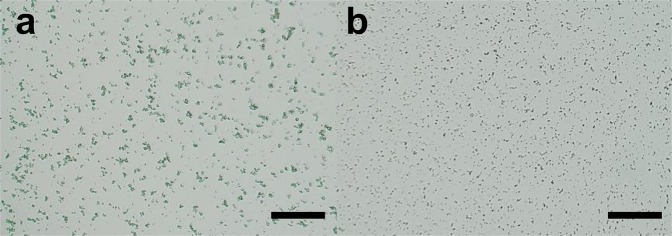
Proteinase K crystals used for SFX measurements, (**a**) sized 2 μm × 2 μm and (**b**) 0.8 μm × 0.8 μm. Scale bars represent 100 μm.

To maintain a continuous and stable sample column by increasing the viscosity of the grease matrix, we developed two types of viscosity-adjustable grease matrices—a paraffin oil-based grease and a DATPE oil-based grease. We collected diffraction images at a wavelength of 1.24 Å using proteinase K crystals sized 2 μm × 2 μm (Fig. 1a) dispersed in the grease matrices. In addition, we performed two data collections using the two conventional matrices^22^—a Super Lube nuclear approved grease (nuclear grease matrix) and a hydroxyethyl cellulose (cellulose matrix)—for comparison of the diffraction data qualities with those of the novel grease matrices. At a flow rate of 0.24 μl min^−1^ through an injector nozzle with an i.d. of 75 μm, we used a total sample volume of about 20 μl with a crystal number density (after mixing with the matrix) of 9.4 × 10^7^ crystals ml^−1^ for 25% (*w/w*) dextrin palmitate/paraffin grease (paraffin grease) and 1.4 × 10^8^ crystals ml^−1^ for 25% (*w/w*) dextrin palmitate/DATPE grease (DATPE grease), nuclear grease and 18% (*w/v*) cellulose matrix (Table 1).

**Table 1:** Crystallographic statistics. Values in parentheses are for the outermost shell.

Matrix	Paraffin grease	DATPE grease	Nuclear grease	Cellulose	Frozen paraffin grease
Crystal size (μm)	2 × 2	2 × 2	2 × 2	2 × 2	2 × 2
Crystal density (crystals ml^−1^)	9.4 × 10^7^	1.4 × 10^8^	1.4 × 10^8^	1.4 × 10^8^	9.4 × 10^7^
Nozzle size i.d. (μm)	75	75	75	75	75
Flow rate (μl min^−1^)	0.24	0.24	0.24	0.24	0.24
HPLC pump pressure (M Pa)	0.8	1.0	0.2	0.6	0.7
**Data collection**					
Space group	P4_3_2_1_2	P4_3_2_1_2	P4_3_2_1_2	P4_3_2_1_2	P4_3_2_1_2
Unit-cell parameter					
*a* = *b* (Å)	68.27	68.23	68.28	68.19	68.28
*c* (Å)	108.22	108.14	108.23	108.16	108.21
Number of collected images	160,000	123,752	125,694	143,934	114,382
Number of hits	32,434	35,588	32,723	30,338	25,835
Number of indexed images	27,298	27,542	23,734	24,328	20,363
Number of total reflections	10,895,546	10,897,238	9,157,696	10,267,269	8,299,878
Number of unique reflections	34,559	34,496	34,574	34,472	34,564
Resolution range (Å)	32.6–1.6 (1.63–1.60)	32.5–1.6 (1.63–1.60)	32.6–1.6 (1.63–1.60)	32.5–1.6 (1.63–1.60)	32.6–1.6 (1.63–1.60)
Completeness (%)	100 (100)	100 (100)	100 (100)	100 (100)	100 (100)
Multiplicity	315 (208)	316 (209)	265 (175)	298 (197)	240 (159)
*R*_split_ (%)^†^	8.7 (96.9)	8.4 (92.6)	9.1 (114.8)	9.6 (173.1)	9.9 (117.2)
CC_1/2_	0.990 (0.427)	0.992 (0.432)	0.989 (0.378)	0.992 (0.227)	0.989 (0.351)
CC_ano_	—	—	—	—	—
<*I*/*σ*(*I*)>	7.7 (1.1)	7.7 (1.2)	7.3 (1.0)	6.5 (0.7)	6.6 (1.0)
**Refinement**					
*R*/*R*_free_ (%)	16.2/18.2	16.1/19.0	16.2/18.6	16.0/18.6	15.8/18.4
PDB code	6k2p	6k2r	6k2s	6k2t	6k2v
**Matrix**	**Paraffin grease (native)**	**DATPE grease (Pr-drivative)**			
Crystal size (μm)	0.8 × 0.8	0.8 × 0.8			
density (crystals/ml)	7.2 × 10^8^	7.2 × 10^8^			
Nozzle size i.d. (μm)	50	50			
Flow rate (μl min^−1^)	0.11	0.11			
HPLC pump pressure (M Pa)	0.8	1.4			
**Data collection**					
Space group	P4_3_2_1_2	P4_3_2_1_2			
Unit-cell parameter					
*a* = *b* (Å)	68.30	68.17			
*c* (Å)	108.20	108.16			
Number of collected images	129,276	137,167			
Number of hits	32,252	36,818			
Number of indexed images	14,624	23,406			
Number of total reflections	4,835,853	7,294,533			
Number of unique reflections	28,952	31,494			
Resolution range (Å)	32.6–1.7 (1.73–1.70)	32.5–1.65 (1.68–1.65)			
Completeness (%)	100 (100)	100 (100)			
Multiplicity	167 (118)	232 (164)			
*R*_split_ (%)^†^	11.9 (94.1)	9.4 (99.9)			
CC_1/2_	0.984 (0.494)	0.990 (0.418)			
CC_ano_	—	0.10 (0.02)			
<*I*/*σ*(*I*)>	5.9 (1.2)	7.0 (1.1)			
**Refinement**					
*R*/*R*_free_ (%)	16.0/18.3	16.1/18.1			
PDB code	6k2w	6k2x			

^†^$${R}_{{\rm{split}}}=1/\sqrt{2}\frac{{\sum }_{hkl}|{I}_{even}-{I}_{odd}|}{1/2\,{\sum }_{hkl}|{I}_{even}+{I}_{odd}|}$$.

We extruded the three grease matrices and a cellulose matrix each as a continuous sample column with a diameter of ~75 μm through a 75-μm-i.d. nozzle (Supplementary Fig. S1a–d). However, in the middle of the data collection experiment for the nuclear grease, we noted a tendency of the matrix to produce an unstable stream with a larger-diameter (~100 μm) grease column (Supplementary Fig. S1e). The viscosity of nuclear grease is substantially lower than that of other matrices (Supplementary Table S1 and Supplementary Figs. S2 and S3), and approximately corresponds to ~15% dextrin palmitate/paraffin or DATPE grease.

### Data collection and crystal structures of proteinase K

**Figure 2:**
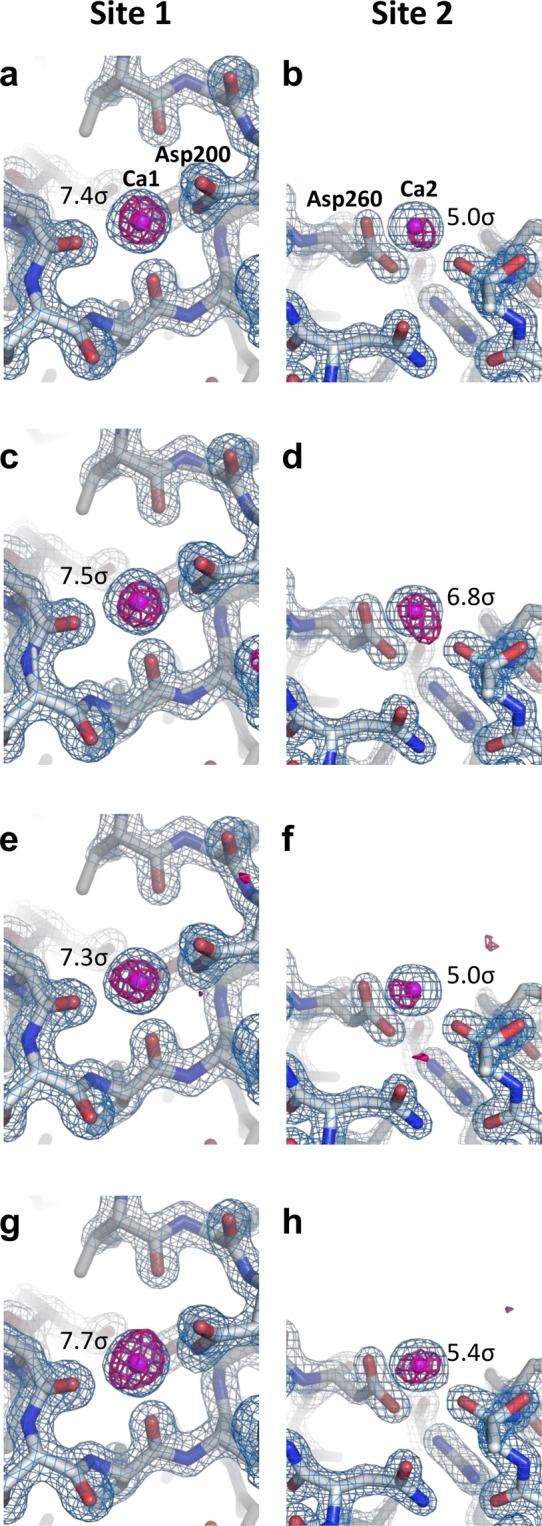
Electron density maps of proteinase K. Close-up views of calcium ion binding sites with 2F_o_–F_c_ electron density maps contoured at the 2.0 σ level (coloured blue) for (**a**,**b**) paraffin grease, (**c**,**d**) DATPE grease, (**e**,**f**) nuclear grease and (**g**,**h**) cellulose matrices. Bound calcium ions are depicted as magenta spheres. The anomalous difference Fourier maps using 20,000 indexed images (contoured at the 3.5 σ level) are shown in red. The anomalous peak heights obtained from ANODE are displayed in figures. These figures were drawn with PyMol (http://www.pymol.org).

We were able to collect ~120,000–160,000 diffraction images in approximately 70 min–90 min with the SACLA running at a 30-Hz repetition rate. We successfully indexed and integrated 24,000–28,000 images for the proteinase K crystals sized 2 μm × 2 μm (space group P4_3_2_1_2). The crystals yielded 100% complete datasets, with a CC_1/2_ ranging from 0.992 to 0.989 at a resolution of 1.6 Å. We determined and refined the four crystal structures of proteinase K at a resolution of 1.6 Å (Table 1). A stronger background scattering in the resolution range of 4 Å–5 Å was visible in the three grease matrices than in the cellulose matrix (Supplementary Fig. S4), and in polysaccharide hydrogels (such as hyaluronic acid and hydroxyethyl cellulose) that have lower background scattering^21,22^. We were able to observe clear electron density maps of proteinase K (Fig. 2). Proteinase K has two binding sites for calcium ions.

### Micro-extrusion of nanocrystals

Using the two novel carriers of the paraffin and DATPE matrices, we attempted the *de novo* phasing of proteinase K (crystal sizes 0.8 μm × 0.8 μm, Fig. 1b) to demonstrate their general applicability as nanocrystal carriers. We extruded the proteinase K nanocrystals with a 25% (*w/w*) dextrin palmitate/paraffin matrix for native and a 25% (*w/w*) dextrin palmitate/DATPE matrix for praseodymium (Pr)-derivative through a 50-μm-i.d. nozzle. We used a flow rate of 0.11 μl min^−1^ for all samples with a crystal number density of 7.2 × 10^8^ crystals ml^−1^. We collected ~130,000–140,000 images from the nanocrystals of native and Pr-derivatised proteinase K—in total, about 8 μl of the matrix volume—at a wavelength of 1.24 Å. In space group *P*4_3_2_1_2, we indexed and integrated approximately 15,000 images for native and 23,000 images for derivative. We used the nanocrystals to acquire datasets at a resolution of 1.7 Å with a CC_1/2_ of 0.984 for native and at a resolution of 1.65 Å with a CC_1/2_ of 0.990 for derivative (Table 1).

**Figure 3:**
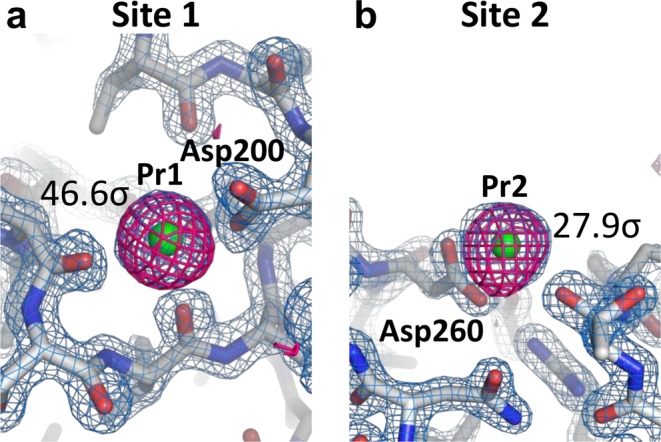
Electron density maps for proteinase K nanocrystals: (**a**,**b**) close-up views of Pr ion binding sites with 2F_o_–F_c_ electron density maps contoured at the 2.0 σ level (coloured blue). Bound Pr ions are depicted as green spheres. The anomalous difference Fourier maps using all indexed images (contoured at the 3.5 σ level) are shown in magenta. These figures were drawn with PyMol (http://www.pymol.org).

Subsequent single-wavelength anomalous diffraction (SAD) phasing, we performed substructure determination and phasing using *SHELXD* and *SHELXE*^43^. We have successfully found in locating two Pr ions in the asymmetric unit and then solved the substructure. We observed the two Pr-binding sites to be identical to those of the calcium ions in the native structure (Fig. 3), indicating that the Pr atoms replaced the two calcium atoms^22,44^. We employed the coordinates of the heavy atoms for both the refinement and the phase calculation at a resolution of 1.8 Å in *SHEXLE*, which also automatically traced a polyalanine model of proteinase K. Subsequently, *Buccaneer*^45^ automatically modelled 99% (276 of 279 residues) of the structure with side chains. Finally, we refined the structure at a resolution of 1.65 Å to an *R*/*R*_free_ of 16.1%/18.1%. As shown in Fig. 3, in the final anomalous difference Fourier maps using all indexed images, we display significant anomalous peak heights (46.6 σ and 27.9 σ, obtained from *ANODE*^46^) of the two Pr atoms. We discuss the structural determination for the *de novo* phasing in detail elsewhere.

### Data collection at room temperature from a frozen grease matrix with proteinase K crystals

Since the sample preparation for SFX is time consuming, more versatile methods like a long-time storage of the protein crystals in the grease matrix at cryogenic temperatures should be indispensable. To this end, we examined to perform a freeze treatment of the proteinase K crystals (sized 2 μm × 2 μm) in a paraffin matrix, and allowing the matrix to return naturally to room temperature from cryo-condition (see Materials and methods), we extruded the paraffin matrix with randomly oriented crystals through an injector nozzle with an i.d. of 75 μm. The result for the data collection is shown in Table 1.

## Discussion

Samples with low viscosity usually induce curling of the stream and adhesion of the matrix to the injector nozzle surface, thereby producing a resultant larger-diameter grease column. Whereas 25% (*w/w*) dextrin palmitate grease matrices—with a viscosity higher than that of the nuclear grease matrix—prevent the stream curling and the attachment to the injector nozzle top. We failed to extrude the matrix at low concentrations of the dextrin palmitate (less than ~15%) from the injector system as a continuous and a stable sample column. Still more, we were also unable to extrude a matrix at a dextrin palmitate concentration of ~30% as it became too hard for micro-extrusion at that concentration. The ideal dextrin palmitate concentration was found to be around 20%–25%.

**Figure 4:**
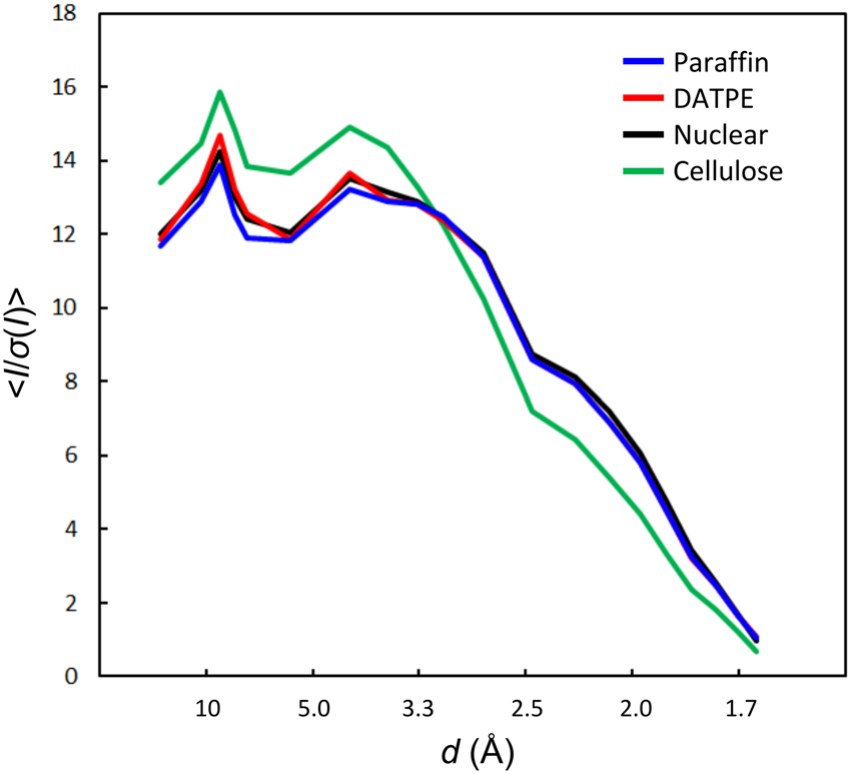
Statistics of I/σ(I) of 20,000 indexed images from each matrix for proteinase K protein. Four datasets of the paraffin, DATPE, nuclear grease and cellulose matrices are coloured in blue, red, black and green, respectively.

We compared the crystallographic statistics from each dataset using 20,000 indexed images (Table 2). We calculated the overall < *I*/*σ*(*I*) > of the merged observations to be 6.6 for paraffin grease, 6.6 for DATPE grease, 6.7 for nuclear grease and 5.9 for cellulose. We found no significant differences in the data collection statistics among the three grease matrices. On the other hand, we noted a tendency of the technique using the cellulose matrix to degrade data quality compared to that of the grease matrices (for instance, the values of *R*_split_ and *I*/*σ*(*I*) in the highest resolution shell of 1.63 Å–1.60 Å). This result might have been due to the cracking and dissolution of protein crystals caused by cellulose media through osmotic shock arising from the properties of hydrogel media^21^. We compared the *I*/*σ*(*I*) for proteinase K using four crystal matrix carriers (Fig. 4). Although we noted slightly higher intensity values of *I*/*σ*(*I*) for the DATPE grease at resolutions ranging from ~10 Å to ~7 Å when compared with those of other grease matrices, we detected no noticeable differences in the statistics for *I*/*σ*(*I*) among the three grease matrices. On the other hand, the statistics of *I*/*σ*(*I*) showed higher intensity values for the cellulose matrix at resolutions ranging from ~17 Å to ~3 Å when compared with those of the grease matrices. However, this trend was reversed on the border of around 3 Å resolution, because a water-based matrix gives a slightly higher background scattering in the resolution range of ~3.5 Å–2.5 Å when compared with the grease matrices^21,22^ (Supplementary Fig. S4).

**Table 2:** Crystallographic statistics for 20,000 indexed images using the data sets from Table 1.

Matrix	Paraffin grease	DATPE grease	Nuclear grease	Cellulose
**Data collection**				
Space group	P4_3_2_1_2	P4_3_2_1_2	P4_3_2_1_2	P4_3_2_1_2
**Unit-cell parameter**				
*a* = *b* (Å)	68.27	68.23	68.28	68.19
*c* (Å)	108.22	108.14	108.23	108.16
Number of indexed images	20,000	20,000	20,000	20,000
Number of total reflections	8,025,924	7,914,751	7,730,797	8,402,679
Number of unique reflections	34,559	34,496	34,574	34,472
Resolution range (Å)	32.6–1.6 (1.63–1.60)	32.5–1.6 (1.63–1.60)	32.6–1.6 (1.63–1.60)	32.5–1.6 (1.63–1.60)
Completeness (%)	100 (100)	100 (100)	100 (100)	100 (100)
Multiplicity	232 (153)	229 (152)	224 (148)	244 (162)
*R*_split_ (%)	10.1 (114.8)	10.1 (109.2)	9.9 (121.9)	10.6 (184.1)
CC_1/2_	0.988 (0.338)	0.988 (0.362)	0.987 (0.349)	0.991 (0.202)
<*I*/*σ*(*I*)>	6.6 (1.0)	6.6 (1.0)	6.7 (0.9)	5.9 (0.6)

Values in parentheses are for the outermost shell.

From 20,000 indexed images of each matrix, we could observe a weak anomalous scattering signal from the calcium atoms in the proteinase K structures (Table 2 and Fig. 2). The averaged anomalous densities for two calcium ions obtained from *ANODE*^46^ were 5.9 σ for paraffin grease, 7.0 σ for DATPE grease, 5.8 σ for nuclear grease and 6.6 σ for cellulose. Among four matrices (Fig. 2a,c,e,g), the anomalous difference Fourier maps for the calcium ion (Ca1) show no noticeable differences, while the signals from the calcium atom (Ca2) are slightly stronger when embedded in the DATPE grease than in the other three matrices (Fig. 2b,d,f,h). These results suggest that the technique using the DATPE grease matrix potentially contributes to the measurement of weak anomalous signals for *de novo* phasing from SFX data. The DATPE grease matrix should accordingly be given higher priority as a grease carrier medium.

For the *de novo* phasing of macromolecules, many studies report having achieved experimental phasing in SFX using heavy-atom derivatives of protein crystals^9,22,35–38,47–50^. We recently performed the *de novo* structural determination of proteinase K from Pr-derivatised crystals (sized 4 μm–7 μm) using a cellulose matrix^22^. The *de novo* phasing was demonstrated using a grease matrix for microcrystals by Hg-SIRAS^36^ and Hg-SAD^37^ for luciferin-regenerating enzyme, Cu-SAD for copper nitrite reductase^35^, I-SAD for bacteriorhodopsin^38^, Se-SAD for the stem domain and galectin^37^ and Gd-MAD (multi-wavelength anomalous diffraction) for lysozyme^50^ at SACLA. Native sulphur SAD phasing was also achieved^34,47,51^. By utilising the anomalous signals of sulphur and chlorine, we have been able, using a grease matrix, to determine the structure of native lysozyme with SAD^34^. These results reinforce the possibility of accurately measuring anomalous signals from nano- and microcrystals using the grease matrix technique.

As a matrix with low background scattering is important in collecting a high-resolution dataset from ~1 μm (or smaller) crystals, the use of a sample column with a smaller diameter (~50 μm), which contributes to the reduction of sample consumption and background scattering from the matrix, is advisable. We therefore extruded the 25% dextrin palmitate/DATPE grease matrix as a continuous column with a diameter of ~50 μm through a 50-μm-i.d. nozzle (Supplementary Fig. S1f), but the 25% dextrin palmitate/paraffin grease had a slightly unstable sample stream with a tendency to induce curling of the stream, due to the lower viscosity of the paraffin grease than that of the DATPE grease at the same concentration of 25% (*w/w*) dextrin palmitate (Supplementary Table S1 and Supplementary Fig. S3). In addition, the pressure values of HPLC pump on sample extrusion in the SFX experiments clearly differentiated the DATPE matrix showed higher pressure than the paraffin matrix (Table 1). This trend of a low viscous sample having an unstable stream was particularly notable when a sample column had a small diameter (such as ~50 μm). Increasing the matrix viscosity by increasing the concentration of dextrin palmitate as a gelator will produce high background scattering noise from the dextrin palmitate. In such cases, a highly viscous DATPE matrix is useful for preventing the disturbance of the sample stream by XFEL exposures in the SFX experiments.

No significant differences in the crystallographic statistics were detected between frozen paraffin (Table 1) and paraffin (for 20,000 indexed images, Table 2) matrices. When we refined the detector metrology by *geoptimiser*^52^ using all indexed images, the r.m.s.d. gave almost identical values of 0.8187 pixels and 0.8186 pixels for the frozen paraffin matrix and for the paraffin matrix, respectively. These results—while they may be limited to the proteinase K crystals (sized 2 μm × 2 μm) used in this study—suggest that this technique will help minimise damage caused by freeze-thaw treatments of crystals in matrices. In our previous study, as a typical and conventional technique for single-crystal X-ray crystallography at ~ −173 °C, we froze a proteinase K crystal (sized ~100 μm) in mother liquor complemented with 30% glycerol as a cryoprotectant^44^. On the other hand, in the SFX study, even though there is no cryoprotectant, small protein crystals (sized, for instance, ~2 μm) may tend to minimise physical damage from ice formation in the protein crystal. By preserving crystals in the matrix at a cryotemperature less than −30 °C, it becomes possible to execute the transportation work (e.g. it would be shipped safely using a dry shipper for cryopreservation) of the crystal samples extremely simply.

In summary, using the two new grease matrices as protein carriers for serial sample loading in SFX, we determined the structures of a proteinase K protein at a resolution of 1.6 Å at room temperature using less than 0.4 mg of sample. We determined the structure at 1.65-Å resolution of the protein in 0.8 μm nanocrystals, applying Pr-SAD phasing to SFX. Carrier media with a stable flow and a small diameter column of the sample have been widely used in various applications in SFX studies—among them, pump-probe studies for light-driven structural changes and chemical dynamics on the femtosecond to millisecond time scale^14–18,53,54^. The technique for serial sample loading using a matrix carrier, which helps in reducing sample consumption, is becoming more important in serial millisecond crystallography using synchrotron radiation^55^. Our grease matrix technique—providing a stable sample flow from a sample injector nozzle—will not only reduce the number of complicated operations during the automated alignment of the sample stream but also make a significant contribution to the development of an automation system for SFX experiments.

## Materials and Methods

### Sample preparation

In this study, we used a proteinase K protein (No. P2308, Sigma) as a protein standard for SFX, because the crystals of proteinase K can assure constant X-ray diffraction quality^21^. In addition, we confirmed that the protein had been successfully derivatized with praseodymium (Pr) ions in our previous studies^22^. We prepared proteinase K (No. P2308, Sigma) crystals sized 2 μm × 2 μm following the protocols reported previously^21^. Nanocrystals (0.8 μm × 0.8 μm) were obtained from a supernatant solution used previously in a crystallisation solution with a batch method. Briefly, for crystals sized 2 μm × 2 μm, 500 μl of 40 mg ml^−1^ protein solution in 20 mM MES–NaOH (pH6.5) were mixed with a precipitant solution composed of 0.5 M NaNO_3_, 0.1 M CaCl_2_, 0.1 M MES–NaOH (pH6.5). As to nanocrystals, they were produced by incubation for 12–24 hours at 18 °C. We filtered the nanocrystal solution through a 5-μm mesh (No. 06-04-0041-2313, CellTrics).

As new grease matrices, we used liquid paraffin oil (128-04375, FUJIFILM Wako Pure Chemical Co.) or dialkyl tetraphenyl ether oil (DATPE) (S-3230, MORESCO) as a base oil, and dextrin palmitate (Rheopearl KL2, Chiba Flour Milling Co.) as a gelator. We kept a 25% (*w/w*) dextrin palmitate/paraffin oil or a 25% (*w/w*) dextrin palmitate/DATPE oil at 110 °C for 3 hours. After filtering the grease through a CellTrics mesh filter (pore size 5 μm), we reheated it to 110 °C for 1 hour. We maintained the grease at room temperature for 2 hours. We ground it with a mortar for 5 min. We describe the preparation protocol for the greases in greater detail in Supplementary Methods.

For the 25% (*w/w*) dextrin palmitate/paraffin matrix, we dispensed a 10-μl crystal sample (sized 2 μm × 2 μm) of storage solution (with a crystal density of 9.4 × 10^8^ crystals ml^−1^) into 90 μl of the paraffin grease on a glass slide and then mixed them with a spatula. For the 25% (*w/w*) dextrin palmitate/DATPE grease and a Super Lube nuclear approved grease (nuclear grease) (No. 42150, Synco Chemical Co.), after centrifuging a 15-μl crystal sample (sized 2 μm × 2 μm) of the storage solution at ~1,300 *g*–3,000 *g* for 10 s with a compact tabletop centrifuge, we removed a 5-μl aliquot of the supernatant solution. We dispensed a 10-μl aliquot of the crystal solution into 90 μl of the grease on a glass slide and then mixed them. We filtered the Super Lube nuclear grade grease through a 10-μm mesh (No. 06-04-0041-2314, CellTrics) before mixing it with protein crystals to remove salt-like impurities in the grease^22^.

For the cellulose matrix, we inserted a 50-μl aliquot of 36% (*w/v*) hydroxyethyl cellulose, 0.5 M NaNO_3_, 0.1 M CaCl_2_ and 0.1 M MES–NaOH (pH 6.5) solution in a 1-ml disposable syringe (SS-01T, Terumo) into a 100-μl Hamilton syringe from the wider end of the syringe, after attaching a syringe coupler (3072–01050, ttplabtech) to the 100-μl syringe. We loaded a 35-μl aliquot of the crystallisation solution into another 100-μl Hamilton syringe (*in this step, it is easy to adjust the cellulose concentration for the matrix viscosity by changing this crystallisation solution volume as necessary*). We connected the syringe for harvest solution to the coupler attached to the syringe for the cellulose matrix, and homogenised the contents of the two syringes by moving the sample back and forth between syringes 50–100 times^24,56,57^. After disconnecting the empty syringe from the coupler, we dispensed 85 μl of the matrix from the syringe onto a glass slide through the coupler. We pipetted a 15-μl crystal sample of storage solution (with a crystal density of 9.4 × 10^8^ crystals ml^−1^) into the matrix and then mixed them with a spatula for 10 s–20 s.

For the proteinase K nanocrystals (0.8 μm × 0.8 μm), after centrifuging a 100-μl crystal sample of storage solution (with a crystal density of 7.2 × 10^8^ crystals ml^−1^) at ~1,300 *g*–3,000 *g* for 10 s with a compact tabletop centrifuge, we removed a 90-μl aliquot of the supernatant solution. For native nanocrystals, we dispensed a 10-μl aliquot of the crystal solution into a 90-μl aliquot of 25% (*w/w*) dextrin palmitate/paraffin grease onto a glass slide and then mixed them. For Pr-derivatised proteinase K, we added a 10-μl sample of the concentrated nanocrystal solution to a 90-μl heavy-atom solution comprising 27.8 mM PrCl_3_ (HR2-450-14, Hampton Research), 0.5 M NaNO_3_ and 0.1 M MES–NaOH (pH 6.5). We then incubated the crystal solution at 20 °C for 90 min. After centrifuging a 100-μl crystal sample of the heavy-atom solution for 10 s, we removed a 90-μl aliquot of supernatant solution. We dispensed a 10-μl aliquot of the crystal solution into 90 μl of 25% (*w/w*) dextrin palmitate/DATPE grease onto a glass slide and then mixed them.

For the frozen proteinase K crystals (sized 2 μm × 2 μm) embedded in the grease matrix, after centrifuging a 10-μl sample of storage solution at ~1,300 *g*–3,000 *g* for 10 s using a compact tabletop centrifuge, we removed a 5-μl aliquot of the supernatant solution. We dispensed a 5-μl aliquot of the crystal solution into 95 μl of 25% (*w/w*) dextrin palmitate/paraffin grease onto a glass slide and then mixed them. After inserting the matrix into a 0.5-ml PCR tube, we centrifuged the sample tube at ~1,300 *g*–3,000 *g* for 10 s with a compact tabletop centrifuge. We inserted the tube into liquid nitrogen (LN_2_), and stored the sample at −30 °C for 2 days. We performed data collection of the crystals in the dextrin palmitate/paraffin matrix after it had been standing for 3 hours at room temperature.

### A micro-extrusion test to estimate matrix viscosity

We performed a simple extrusion test to estimate suitable matrix viscosity for a serial sample loading using 15%–25% (*w/w*) dextrin palmitate/paraffin grease, 15%–25% (*w/w*) dextrin palmitate/DATPE grease, nuclear grease and 18% (*w/v*) cellulose solution [0.5 M NaNO_3_, 0.1 M CaCl_2_ and 0.1 M MES–NaOH (pH 6.5)]. We loaded the matrix into a 100-μl Hamilton syringe, and manually extruded the matrix through a mosquito LCP needle (inner diameter ~430 μm, 4150–05902, ttplabtech). We measured the maximum length of the continuous matrix column until the stream is cut off, using a scale (Supplementary Fig. S2). We repeated each measurement at least three times and took the average of all measurements.

We performed viscosity measurements using a Kinexus rheometer (Malvern). The viscosity of each matrix was measured at 25 °C using a cone plate geometry (CP2/20 SR2254SS) with shear rates of 0.1, 1.0 and 10 s^−1^. Experiments were carried out in three or more replicates. The results are summarized in Supplementary Table S1.

### Data collection

We carried out the experiments at SACLA^4^ using femtosecond X-ray pulses at a wavelength (photon energy) of 1.24 Å (10 keV) with a pulse energy of ~ 200 μJ. Each X-ray pulse delivers ~10^11^ photons within a 10-fs duration (FWHM) to the sample. The X-ray beam was focused with Kirkpatrick-Baez mirrors^58^ to have a spot size of 1.5 × 1.5 μm^2^. The crystals in the matrix were serially supplied using a high viscosity micro-extrusion injector system in a helium ambiance^59^. We extruded the matrix with randomly oriented crystals through injector nozzles with an inner diameter (i.d.) of 75 μm or 50 μm (Table 1) by using an HPLC system (LC-20AD, Shimadzu). The crystals were kept at approximately 20 °C in the injector. We collected data using a diverse application platform for hard X-ray diffraction in SACLA (DAPHNIS) at BL2^60,61^. Diffraction images were recorded using a custom-built 4 M-pixel detector with multi-port CCD sensors^62^. Real-time data analysis were performed via the SACLA data processing pipeline^63^.

### Background intensity determination

We determined the background intensities from paraffin grease, DATPE grease, nuclear grease and cellulose through a 75-μm-i.d. nozzle at 1.24 Å by a procedure similar to that used in Conrad *et al*.^24^ We calculated the average (*m*) and standard deviation (*s*) of each detector pixel over images. To remove intensity contributions due to Bragg spots from protein crystals, we rejected pixels brighter than *m* + 3 *s*. Remaining pixels were averaged again to yield a “clean” background image^21^.

### Structure determination

We filtered and converted the diffraction images using *Cheetah*^64^, adapted for the SACLA data acquisition system^63,65^. We determined diffraction peak positions using the built-in Zaefferer^66^ or the peakfinder8^64^ algorithm and passed them on to *DirAx*^67^ or *asdf* for indexing. We applied no sigma cutoff or saturation cutoff. We merged measured diffraction intensities by *process_hkl* in the *CrystFEL* suite^68^ with scaling (–*scale* option). We determined the structures of proteinase K by difference Fourier synthesis using a search model (PDB: 5b1d). For the Pr-derivatised proteinase K, we carried out substructure search, phasing and phase improvement using the *SHELX C, D* and *E* programmes^43^. We fed the auto-traced model from *SHELXE* into *Buccaneer*^45^ from the *CCP4* suite^69^. We performed manual model revision and structure refinement using *Coot*^70^ and *PHENIX*^71^, respectively. In Table 1, we summarise details of the data collection and refinement statistics.

## Supplementary information

Supplementary information.
