# Supplementary material for: Viscosity-adjustable grease matrices for serial nanocrystallography
Source: Sci Rep. 2020 Jan 28;10:1371. doi: 10.1038/s41598-020-57675-7 (PMC6987181; doi:10.1038/s41598-020-57675-7)
Supplement: Supplementary file 1 — Supplementary information. [file 41598_2020_57675_MOESM1_ESM.pdf]

Viscosity-adjustable grease matrices for serial nanocrystallography

Michihiro Sugahara, Koji Motomura, Mamoru Suzuki, Tetsuya Masuda, Yasumasa Joti, Keiji Numata, Kensuke Tono, Makina Yabashi, Tetsuya Ishikawa

Supplementary information

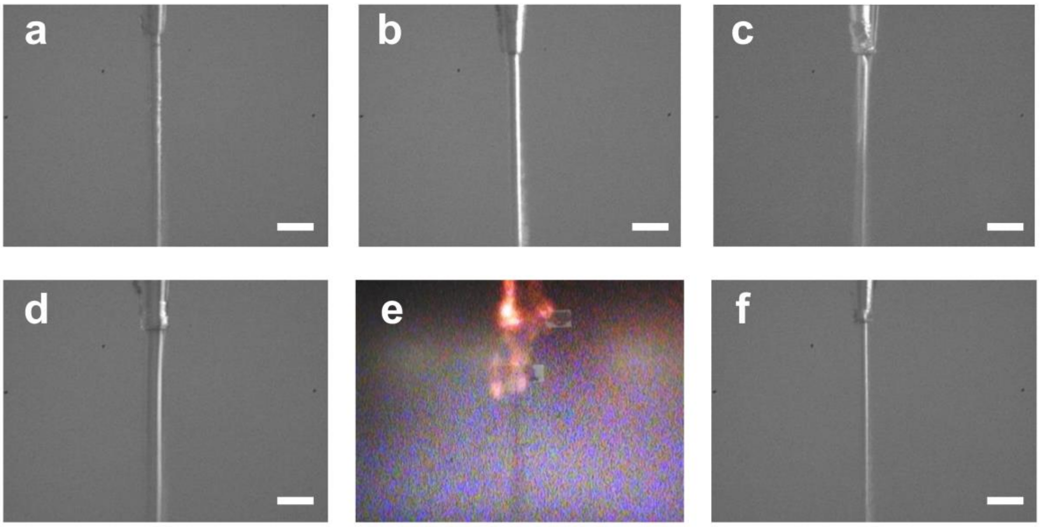

Supplementary Fig. S1

Sample extrusion of the four matrices: (a) Paraffin grease, (b) DATPE grease, (c) nuclear grease and (d) cellulose matrices were extruded through an injector nozzle with an i.d. of 75 µm. (e) An unstable stream for nuclear grease through a 75-µm-i.d. nozzle. (f) Micro-extrusion for DATPE grease through a 50-µm-i.d. nozzle. Scale bars represent 200 µm.

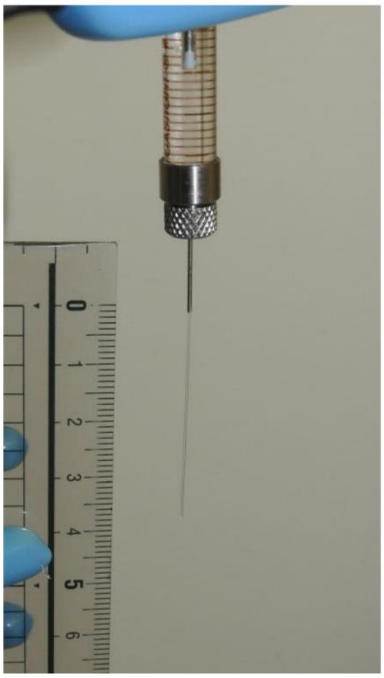

Supplementary Fig. S2

Micro-extrusion test to evaluate matrix viscosity.

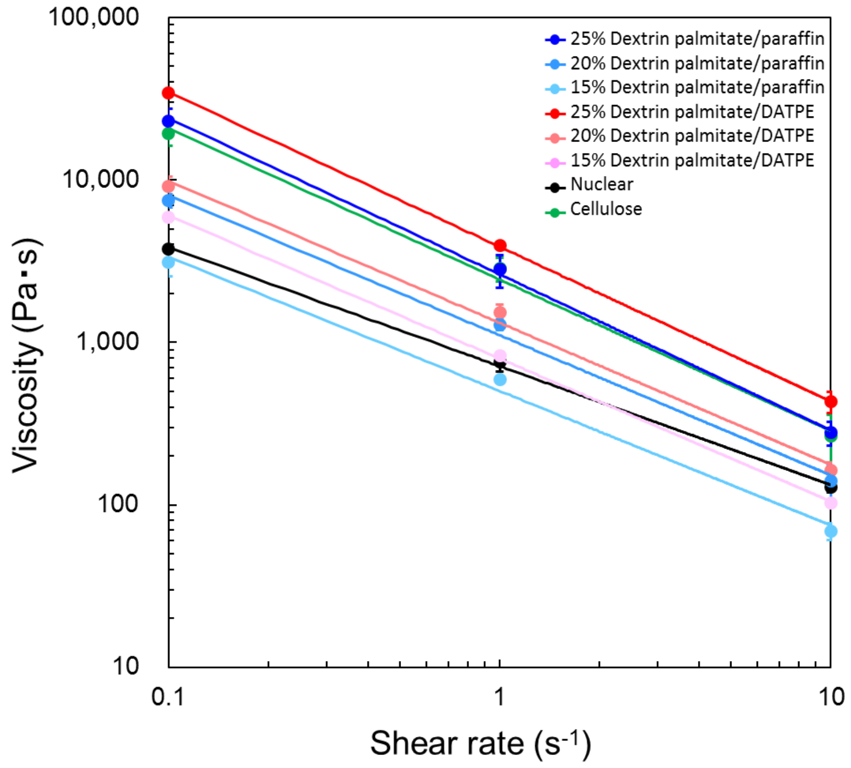

Supplementary Fig. S3

Viscosity as a function of shear-rate for each matrix. Paraffin grease, DATPE grease, nuclear grease and cellulose are depicted by the blue, red, black and green lines, respectively.

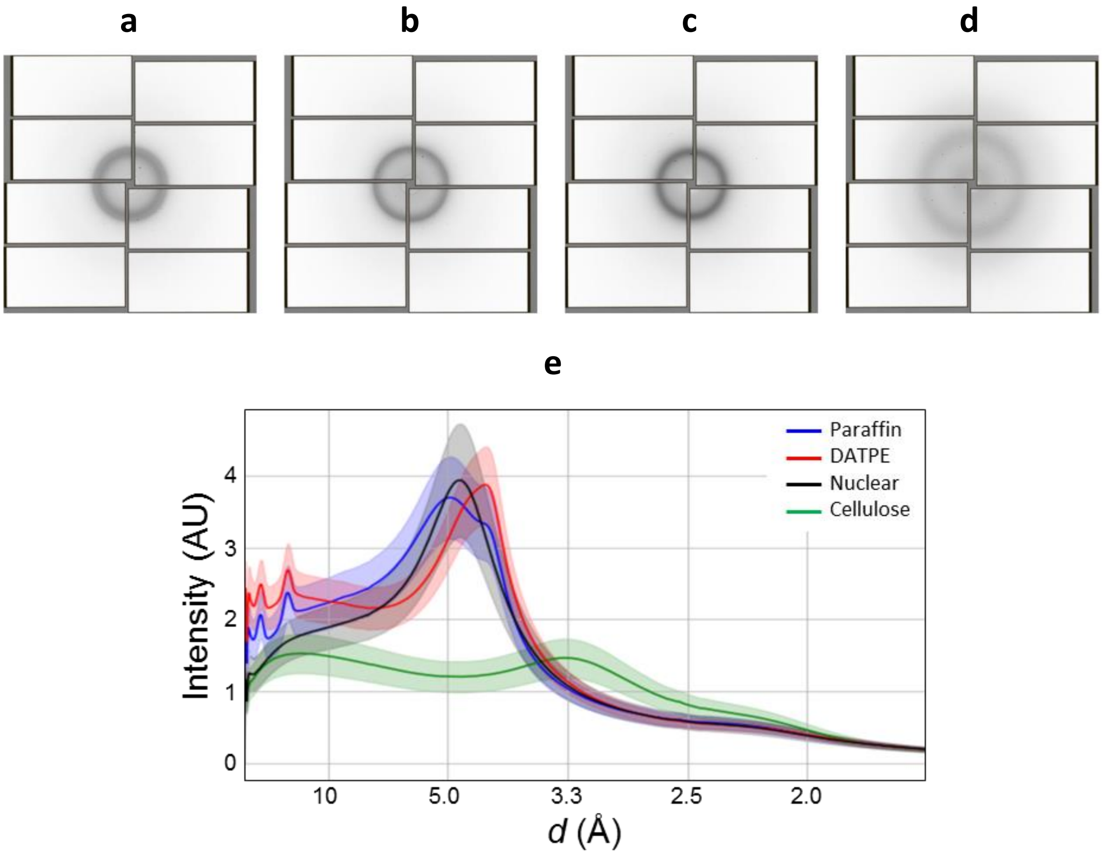

Supplementary Fig. S4

Typical XFEL single diffraction images: (a) paraffin grease, (b) DATPE grease, (c) nuclear grease and (d) cellulose matrices. (e) The average background scattering intensities of ~10,000 images from each matrix. Paraffin grease, DATPE grease, nuclear grease and cellulose are depicted by the blue, red, black and green lines, respectively. The error or fluctuation in the intensity is shown as transparent regions.

Supplementary Methods

A preparation method for paraffin and dialkyl tetraphenyl ether (DATPE) greases

Materials

Base oil

● liquid paraffin (128-04375, FUJIFILM Wako Pure Chemical Co.)

● dialkyl tetraphenyl ether (DATPE) (S-3230, MORESCO)

Gelator

● dextrin palmitate (Rheopearl KL2, Chiba Flour Milling Co.)

Methods

Paraffin and DATPE greases (required time: each 7 hours)

Paraffin grease: 25% (*w/w*) dextrin palmitate in paraffin oil

● colour: colourless transparent grease

DATPE grease: 25% (*w/w*) dextrin palmitate in DATPE oil

● colour: light yellow transparent grease

● This grease has a higher viscosity than 25% (*w/w*) dextrin palmitate/paraffin grease

1| For paraffin oil, place 7,500 mg of paraffin oil and 2,500 mg of dextrin palmitate (weight ratio, paraffin oil : dextrin palmitate = 3 : 1) into a 15-ml conical tube. Gently mix with a spatula.

For DATPE oil, grind the mixture of 7,500 mg DATPE oil and 2,500 mg dextrin palmitate using a mortar for 5 min (**Supplementary Fig. S5**).

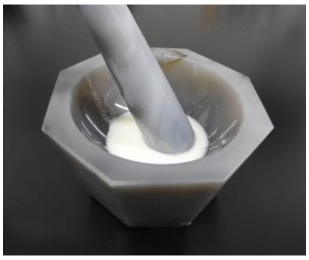

Supplementary Fig. S5

Mixing preparation for dextrin palmitate and DATPE oil using a mortar.

2| Divide into 1.5-ml disposable tubes.

3| Heat for 3 hours at 110 °C (**Supplementary Fig. S6**). Perform vortex mixing approximately once per hour.

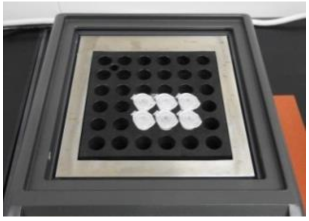

Supplementary Fig. S6

The mixture is heated to 110 °C.

4| Filter the grease through a mesh with a pore size of 5 μm (06-04-0041-2313, Celltrics) using a plunger assembly of a 5-ml disposable syringe while it is hot, to remove impurities in the grease (**Supplementary Fig. S7**).

a **b**

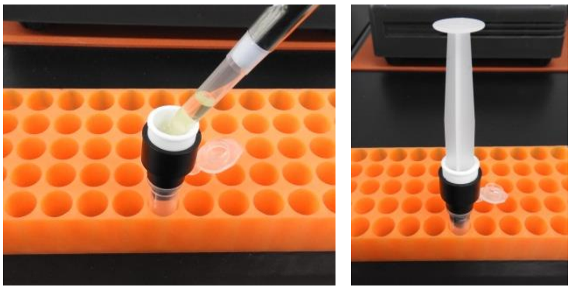

Supplementary Fig. S7

Grease filtration. The grease is dispensed into the Celltrics filter (a) and extruded by pushing the plunger (b).

5| Heat for 1 hour at 110 °C.

6| Store the grease for over 2 hours at room temperature (~22 °C).

7| Grind the grease using a mortar for 5 min (**Supplementary Fig. S8**). By grinding the greases in this step, the greases acquire the property of producing a continuous sample column. If you observe impurities in the grease under a Hirox digital microscope or a polarizing microscope, the process moves back to Step 3.

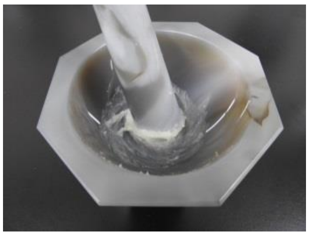

Supplementary Fig. S8

The grease is ground using a mortar.

8| Insert the grease matrix into a 5.0- or 10-ml disposable syringe. Store the matrix at room temperature.

Supplementary Table S1

Micro-extrusion test to evaluate matrix viscosity.

Matrix	Paraffin grease			DATPE grease			Nuclear grease	Cellulose
Dextrin palmitate concentration [%(*w*/*w*)]	15	20	25	15	20	25	0	0
Continuous column length (cm)	6 ± 1	18 ± 4	35 ± 7	6 ± 1	27 ± 4	more than 45	8 ± 1	35 ± 5
Column diameter (μm)	410 ± 32	401 ± 33	427 ± 44	382 ± 41	412 ± 30	417 ± 37	432 ± 20	488 ± 44
**Viscosity measurements**								
Viscosity at a shear rate of 0.1 s^{-1} (Pa·s)	3,112 ± 559	7,467 ± 667	23,137 ± 4,226	5,910 ± 241	9,158 ± 1,269	34,497 ± 572	3,743 ± 164	19,229 ± 2,938
Viscosity at a shear rate of 1.0 s^{-1} (Pa·s)	589 ± 18	1,287 ± 91	2,811 ± 631	827 ± 36	1,531 ± 173	3,932 ± 163	754 ± 92	2,847 ± 471
Viscosity at a shear rate of 10 s^{-1} (Pa·s)	69 ± 8	141 ± 35	278 ± 47	103 ± 4	164 ± 19	430 ± 63	129 ± 10	264 ± 95
